# Concomitant Presence of *Aspergillus* Species and *Mycobacterium* Species in the Respiratory Tract of Patients: Underestimated Co-occurrence?

**DOI:** 10.3389/fmicb.2019.02980

**Published:** 2020-01-10

**Authors:** Sarah Dellière, Cécile Angebault, Vincent Fihman, Françoise Foulet, Raphaël Lepeule, Bernard Maitre, Frédéric Schlemmer, Françoise Botterel

**Affiliations:** ^1^Unité de Parasitologie – Mycologie, Département de Virologie, Bactériologie-Hygiène Mycologie-Parasitologie, Unité Transversale du Traitement des Infections (VBHMP – UT2I), DHU VIC, 75 APHP, CHU Henri Mondor, Créteil, France; ^2^Dynamyc, UPEC, EnVA, ANSES, Créteil, France; ^3^Unité de Bactériologie – Mycologie, Département de Virologie, Bactériologie-Hygiène Mycologie-Parasitologie, Unité Transversale du Traitement des Infections (VBHMP – UT2I), DHU VIC, 75 APHP, CHU Henri Mondor, Créteil, France; ^4^Département de Virologie, Bactériologie-Hygiène Mycologie-Parasitologie, Unité Transversale du Traitement des Infections (VBHMP – UT2I), DHU VIC, 75 APHP, CHU Henri Mondor, Créteil, France; ^5^Service de Pneumologie, DHU A-TVB, APHP, CHU Henri Mondor, Créteil, France

**Keywords:** *Aspergillus* sp., *Mycobacterium* sp., chronic aspergillosis, tuberculosis, non-tuberculous mycobacteria

## Abstract

**Objectives:**

*Aspergillus* and *Mycobacterium* are opportunistic pathogens that can cause severe pulmonary diseases. To date, the clinical significance of their concomitant isolation and potential interactions in the lung remains poorly understood. The aim of this study was to assess the prevalence of their concomitant isolation from respiratory samples, and to depict the related clinical and microbiological characteristics.

**Methods:**

A retrospective monocentric study was conducted from January 2011 to December 2017, including all in-patients from whom positive cultures of *Aspergillus* and *Mycobacterium* were obtained on respiratory samples within a 3-month period. Clinical, radiological and laboratory data were analyzed. Patients were categorized by a clinical and microbiological committee as “infected” or “colonized” by both pathogens according to current guidelines.

**Results:**

Overall, 140 patients had ≥1 respiratory samples positive for *Mycobacterium* and concomitantly sent for fungal culture, and 708 were positive for *Aspergillus*, concomitantly sent for mycobacterial culture. Only 50 had at least one positive culture for both *Mycobacterium* sp. and *Aspergillus* sp. Men represented 63% of patients, mean age was 61 years. A third of patients were immunocompromised and 92% had underlying lung diseases. *Aspergillus* was primarily found as a colonizing agent. Proportion of *Mycobacterium Avium* Complex (*p* = 0.02) was higher in patients co-carrying *Aspergillus* spp.

**Conclusion:**

In this first study focusing on co-isolation of *Mycobacteria* and *Aspergillus* in patient’s respiratory samples, co-infection remains rare. Further studies are warranted in order to precise the exact relationship between these opportunistic pathogens and the clinical impact of co-isolations.

## Introduction

*Mycobacteria* are commonly divided into *Mycobacterium tuberculosis* complex, which causes tuberculosis (TB), and non-tuberculous *Mycobacteria* (NTM), which cause opportunistic lung diseases in patients with pre-existing pulmonary disease and/or mild to severe immunosuppression. Over the past three decades, NTM detection has increased in clinical laboratory, probably due to both improved culture techniques and the actual increase in NTM incidence (Johnson and Odell, 2014; Kontturi et al., 2018). Unlike TB, the detection of NTM in pulmonary specimen does not always denote the disease itself since NTM are common environmental germs. Precise and worldwide epidemiology data on NTM prevalence are lacking (Lin et al., 2018). Therefore, the American Thoracic Society (ATS), the Infectious Diseases Society of America (IDSA), and more recently the British Thoracic Society have elaborated guidelines on the management of NTM lung diseases to help clinicians in routine practice (Griffith et al., 2007; Haworth et al., 2017).

*Aspergillus* is a ubiquitous mold that causes invasive pulmonary aspergillosis (IPA) and various clinical and radiological forms of chronic pulmonary aspergillosis (CPA) in patients with pre-existing pulmonary diseases (Kousha et al., 2011). CPA like simple pulmonary aspergilloma (SPA), chronic cavitary pulmonary aspergillosis (CCPA), and subacute invasive aspergillosis (or necrotizing CPA) are associated with different degrees of lung damage (Denning et al., 2016). Hypersensitivity to *Aspergillus fumigatus* can lead to another form of aspergillosis called allergic broncho-pulmonary aspergillosis (ABPA) (Agarwal et al., 2013).

Given that both NTM and *Aspergillus* are ubiquitous environmental organisms often isolated from patients with other lung diseases, it might be difficult sometimes to differentiate colonization from active infection caused by one of these organisms. In both cases, the treatment is often long, poorly tolerated, requiring antimicrobials with many side effects and drug interactions, and in some cases surgical lung resection. Recent studies highlighted that aspergillosis can occur in patients with NTM lung disease, which worsens the prognosis and creates several therapeutic issues (Kobashi et al., 2006; Zoumot et al., 2014; Takeda et al., 2016; Furuuchi et al., 2017). A significant increase in mortality has been described in patients with NTM lung disease when *A. fumigatus* was also present (Furuuchi et al., 2017).

For such, clinical cases wherein *Mycobacterium* and *Aspergillus* are concomitantly isolated from respiratory samples constitute a complex puzzle. In both cases, clinical symptoms are non-specific and CT-scan abnormalities can be attributed to one or both pathogens with no particular features (Griffith et al., 2007; Denning et al., 2016). Rigorous evaluation of each case seems essential to avoid misdiagnosis, therapeutic delay, or unnecessary administration of long and potentially deleterious therapeutics. In order to help physicians to unravel such challenging clinical situations, we designed this study to depict the clinical, radiological, and microbiological characteristics, the management and the outcome of patients who had *Aspergillus* and *Mycobacterium* concomitantly isolated from their respiratory samples.

## Patients and Methods

This retrospective study was conducted from January 2011 to December 2017 in a tertiary care institution (Henri Mondor University Hospital, Créteil, France). Patients were enrolled if they matched two databases, both extracted from the logs of the microbiology laboratory. The first database included patients who had at least one respiratory sample [sputum, tracheal aspirate, protected sample brush, bronchoalveolar lavage (BAL)] yielding positive *Aspergillus* spp. culture during the study period. The second included patients with *Mycobacterium* spp. detected in at least one respiratory sample. Patients who had *Aspergillus*- and *Mycobacterium*-positive cultures separated by more than a 3-month period were excluded to strictly focus on co-isolation cases.

**FIGURE 1:**
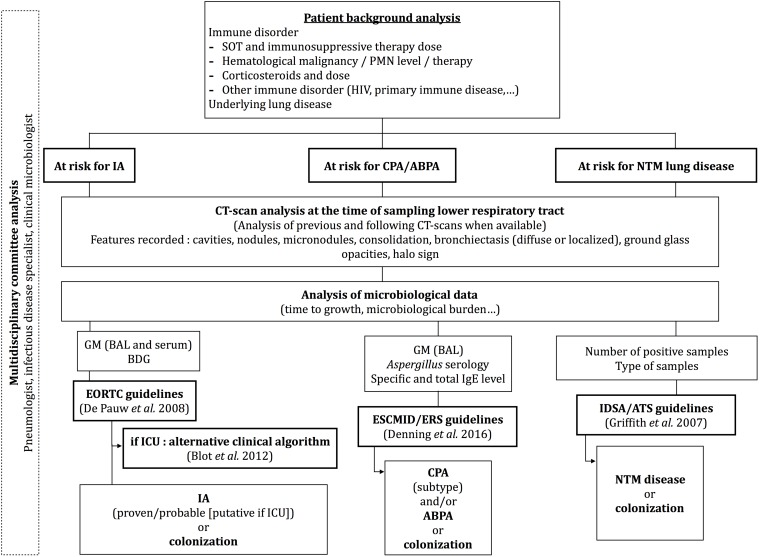
Algorithm used by a multidisciplinary committee to classify patients as infected or colonized by *Aspergillus* or NTM. ABPA, allergic bronchopulmonary aspergillosis; BDG, ß-1,3-glucan; CPA, chronic pulmonary aspergillosis; GM, galactomannane; IA, invasive aspergillosis; NTM, non-tuberculous mycobacteria; PMN, polymorphonuclear; SOT, solid organ transplant.

When non-tuberculous mycobacterium (NTM) was isolated, cases were classified as colonization or infection according to American Thoracic Society (ATS)/ Infectious Diseases Society of America (IDSA) criteria (Griffith et al., 2007; Figure 1). When *M. tuberculosis* complex was isolated, the diagnosis of pulmonary TB was established. Regarding *Aspergillus*, cases were classified as CPA according to ESCMID guidelines (Denning et al., 2016) or IPA according to EORTC criteria for immunocompromised patients or Blot modified criteria for critically ill patients (De Pauw et al., 2008; Blot et al., 2012; Ullmann et al., 2018; Levesque et al., 2019). If patients were not included in these definitions, they were classified as colonized (Figure 1). Finally, both microbiologist and pulmonologist reviewed the medical charts of all of these patients to analyze clinical, radiological, and microbiological data. *Aspergillus* serology combining IgG detection (Platelia *Aspergillus* IgG, Bio-Rad, France) and *Aspergillus* precipitins (*Aspergillus* immunodiffusion system, Microgen Bioproducts, United Kingdom and Hydragel IEP Plus, France) were collected whenever available. Positive serology comprises an ELISA titer >10 IU/mL and a precipitin test with ≥2 precipitin lines according to the manufacturer instructions. Standard bacterial cultures from respiratory specimens were also collected in the same 3-month period.

Clinical and microbiological characteristics of the population were reported in percentage, mean and standard deviation (SD) as appropriate. Clinical and microbiological characteristics of patients with and without concomitant *Aspergillus* spp.*/Mycobacterium* spp. colonization were compared using Pearson chi-square or Fisher test for qualitative variables and Student’s *t*-test or Wilcoxon-Mann-Whitney for quantitative variables as appropriate. The data were analyzed using Prism 6.0 software.

The authors testify that all procedures contributing to this work are in compliance with the ethical standards of the Helsinki Declaration of 1975, as revised in 2008. Since the study was retrospectively conducted on isolates collected through routine clinical work and patient’s identifiable information had already been anonymized, no written or verbal informed consent was necessary for patients to participate in this study.

## Results

**FIGURE 2:**
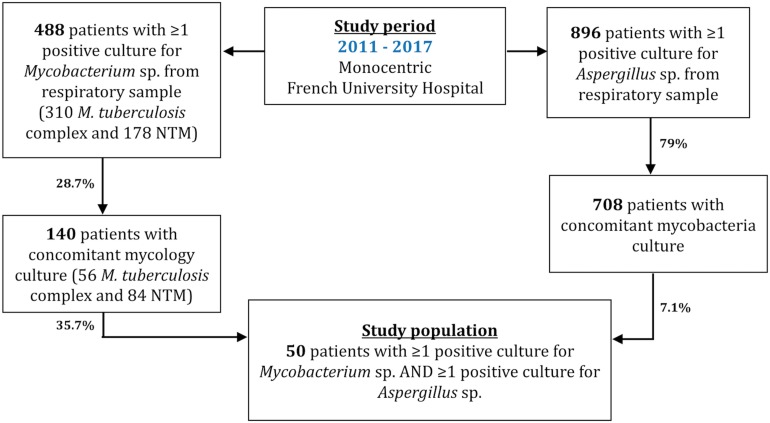
Flow chart of the monocentric retrospective study conducted between January 2011 and December 2017. Patients were included with positive *Aspergillus* sp. and *Mycobacterium* culture from lower respiratory tract samples. Concomitant cultures define cultures performed on a respiratory sample within less than 3 months apart.

During the study period, 488 and 896 patients had at least one respiratory sample positive for *Mycobacterium* sp. and *Aspergillus* sp., respectively. Of those, 140/488 (28.7%) patients had their *Mycobacteria*-positive sample concomitantly sent to the mycology department for culture, and 708/896 (79%) had their *Aspergillus*-positive sample concomitantly searched for *Mycobacteria*. A total of 50 patients had at least one positive culture for both *Mycobacterium* sp. and *Aspergillus* sp. (Figure 2). In another word, of those with positive *Mycobacterium*, 35.7% (50/140) carried concomitant *Aspergillus*, and 7.1% (50/708) patients with positive *Aspergillus* were concomitantly carrying *Mycobacterium* (Figure 2). Of note, simultaneous discovery of both organisms was observed in 24 patients, whereas for the remaining 26 patients, *Mycobacterium* was isolated first in 18 of them. Men represented 63% of patients and mean age was 61 years. A third of patients were immunocompromised and 92% (*n* = 46) had underlying lung diseases, mostly in the form of bronchiectasis (64%). Patients’ characteristics and CT scan abnormalities are described in Table 1.

**TABLE 1:** Clinical and radiological characteristics of 50 patients with concomitant detection of *Aspergillus* sp. and *Mycobacterium* sp. in respiratory samples.

**Characteristics**	***n* (%) Total *n* = 50**
**Sex ratio M/F**	1.63
**Age (± SD) (years)**	61 (± 8)
**Smokers (active or former)**	22 (44)
**Underlying lung disease**	46 (92)
COPD	7 (14)
Emphysema	7 (14)
Bronchiectasis	32 (64)
Asthma	5 (10)
ABPA	1 (2)
Interstitial lung disease	4 (8)
Neoplasia	2 (4)
**Corticosteroid use (prednisone-equivalent)**	26 (52)
<10 mg/day	3 (6)
≥10 mg/day	12 (24)
Inhaled	11 (22)
**Immunocompromised population**	18 (36)
Solid Organ Transplant	7 (14)
Kidney	5 (10)
Heart	1 (2)
Liver	1 (2)
Blood malignancies	7 (14)
HIV	4 (8)
Primary immune disorder	1 (2)
**CT scan abnormalities**	50 (100)
Bronchiectasis	32 (64)
Localized	17 (34)
Diffuse	15 (30)
Nodules	18 (36)
Micronodules	36 (72)
Cavities	4 (8)
Consolidation	14 (28)
Ground glass opacities	3 (6)
None	0 (0)

*ABPA, Allergic Bronchopulmonary Aspergillosis; COPD, chronic obstructive pulmonary disease; CT, Computed tomography; HIV, Human Immunodeficiency Virus.*

The *Aspergillus* species isolated from the first positive respiratory sample were *Aspergillus Fumigati* (*n* = 33), *Aspergillus Nigri* (*n* = 6), *Aspergillus Terrei* (*n* = 1), *Aspergillus Flavi* (*n* = 1), *Aspergillus versicolor* clade (*n* = 2), *Aspergillus chevalieri* clade (*n* = 1), and one *Aspergillus* sp. (without molecular identification). Five patients had multiple *Aspergillus* sp. in the same sample. For the isolated *Mycobacterium* spp., *Mycobacterium avium* complex represented (MAC, *n* = 19), *Mycobacterium tuberculosis complex* (*n* = 14), *Mycobacterium xenopi* (*n* = 8), *Mycobacterium fortuitum* (*n* = 4), *Mycobacterium gordonae* (*n* = 2), *Mycobacterium kansasii* (*n* = 1), *Mycobacterium chelonae* (*n* = 1), and *Mycobacterium abscessus* (*n* = 1). The distribution of *Mycobacterium* sp. was different in patients concomitantly carrying *Aspergillus* versus non-carriers. The proportion of MAC was higher in co-colonized patients (38 vs. 20%, *p* = 0.02), unlike *M. tuberculosis* complex, which was underrepresented in this population (28 vs. 46.7%, *p* = 0.03). Standard bacterial cultures were positive in only 18 of the 50 patients (36%), mostly as *Haemophilus influenzae* (*n* = 8; 16%).

**FIGURE 3:**
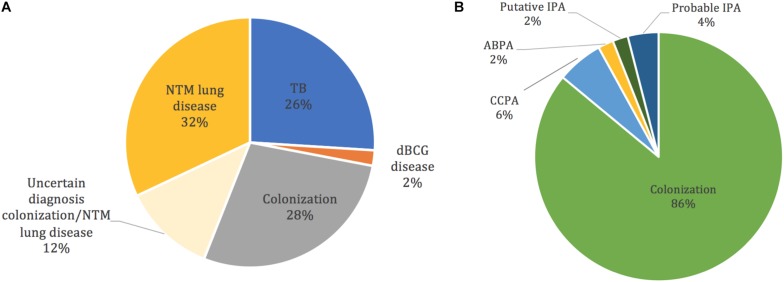
Classification of lung infection or colonization by *Mycobacterium* spp. and *Aspergillus* spp. co-isolated in respiratory samples of 50 patients. **(A)** Pathogenicity of *Mycobacterium* spp.; *M. chelonae*, *M. gordonae*, and *M. fortuitum* are found in the colonization group only. **(B)** Pathogenicity of *Aspergillus* spp. IPA, invasive pulmonary aspergillosis; CCPA, chronic cavity pulmonary aspergillosis; ABPA, allergic bronchopulmonary aspergillosis. dBCG, BCG disease.

The pathogenicity of co-isolated *Mycobacterium* sp. and *Aspergillus* sp. is shown in Figures 3A,B. Of the 14 patients with *M. tuberculosis* complex, 13 had TB and one had BCG disease. Regarding the 36 patients with NTM, 16 had diagnostic criteria for NTM lung disease, 14 were considered as colonized, and 6 remained unclassified even after applying ATS/IDSA guidelines (Figure 3A). *M. chelonae*, *M. gordonae*, and *M. fortuitum* were found in the colonization group only. In patients who carried *Aspergillus*, only seven were diagnosed with CPA (*n* = 3), acute aspergillosis (*n* = 3), and ABPA (*n* = 1) and the causative agent was *Aspergillus fumigatus sensu stricto*. The 43 other patients were classified as colonized (Figure 3B). *Aspergillus* serology was available in 35 (70%) patients. In patients diagnosed with CCPA (*n* = 3), one had positive serology, and two had negative test because the latter patients were on immunosuppressive treatments for renal transplantation. The patient with ABPA had positive serology. Of the patients classified as colonized (*n* = 43), 31 (72%) had serology tests and all were negative.

Anti-tuberculosis treatment was initiated in all patients with *M. tuberculosis* complex. However, only 12 (75%) of the patients diagnosed with NTM lung disease (*n* = 16) were treated. Colonized patients did not receive any treatment (Supplementary Table S1). The comparison of patients with *M. tuberculosis* complex versus NTM is available in Supplementary Table S2. The outcome was similar in the TB or NTM groups (Supplementary Table S2).

In our population, only three (6%) patients were treated both for aspergillosis and mycobacterial infection and were on corticosteroids treatment at the time of diagnosis. Details are shown in Supplementary Table S3.

## Discussion

During our seven-year retrospective study, we identified 50 patients from whom both *Mycobacterium* and *Aspergillus* were co-isolated in respiratory samples during a predefined 3-month period. The prevalence of concomitant *Aspergillus* isolation was high (50/140, 35.7%) when *Mycobacterium* was identified and a fungal culture was concomitantly performed. More specifically, *Aspergillus* was concomitantly detected with *M. tuberculosis* or NTM in 25% (14/56) and 42.9% (36/84), respectively. For comparison, the prevalence of *Aspergillus* isolation was only 12.2% in a Japanese population with MAC lung disease (Furuuchi et al., 2017). The higher co-isolation level we observed could be attributed to the probable selection of patients who underwent a concomitant fungal culture of their respiratory samples, and to the quite high percentage of immunocompromised patients in our study population. Furthermore, the relatively high level of co-isolation observed in NTM patients, as compared with that seen in TB patients at diagnosis, might be due to the higher prevalence of bronchiectasis in NTM patients. In other work, bronchiectasis was described as a major risk factor of NTM infection (Andrejak et al., 2013) and several publications have also underlined the potential burden of *Aspergillus* colonization/infection on CF (cystic fibrosis) and non CF bronchiectasis patients (Amin et al., 2010; Máiz et al., 2015). In both cases, the impaired mucociliary clearance, mucus plugging, and the subsequent local host defenses impairment may explain the persistence of these microorganisms in the respiratory tract of bronchiectasis patients, and their potential pathogenicity. Of note, all of our seven cases of pulmonary aspergillosis (probable IPA, *n* = 2; putative IA, *n* = 1; CPA, *n* = 3; ABPA, *n* = 1) were caused by *A. fumigatus stricto sensu*, which is consistent with the well-known pathogenicity of this species. The results of Furuuchi et al. (2017) as well as the epidemiological data of CPA in Europe show that *A. fumigatus* was also the only *Aspergillus* species detected in CPA in such patients (Cadranel et al., 2012).

Despite our immunocompromised population (*n* = 18), only two probable IPA were observed according to EORTC criteria (De Pauw et al., 2008). Only one patient was classified as a case of putative IPA according to Blot et al. (2012) modified criteria. The other patients have been considered as colonized because radiological signs evocative of aspergillosis (i.e., halo sign, air crescent sign) were not found.

In this population with concomitant presence of *Mycobacterium* and *Aspergillus*, we observed rare cases of patients where the two microorganisms were acting as co-infective pathogens. Classification of both pathogens status was challenging, however, the in-depth examination of the available radiological and microbiological data (including *Aspergillus* serology and BAL analysis for CPA classification) allowed us to distinguish colonization from infection in most cases. Additionally, a positive culture of *Mycobacterium* associated with positive *Aspergillus* antibodies despite a negative fungal culture could also be considered as co-infection. Unfortunately, *Aspergillus* serology data were often missing and for such we estimate that co-infection rate might have been underestimated. Thus, our study underlines the importance of performing, as possible, all available and recommended direct and indirect investigations to optimize the diagnosis and follow-up of potentially co-infected patients. Given that therapeutic decisions for NTM lung infections or aspergillosis are usually discussed by multidisciplinary team, cases with concomitant NTM-Asp infection/occurrence could particularly benefit from such collaboration. Recent studies confirmed that chronic aspergillosis could worsen the outcome of patient already infected with NTM (Zoumot et al., 2014; Furuuchi et al., 2017). Therefore, patients infected (or colonized) by NTM and colonized by *Aspergillus* may require a close follow-up in order to detect CPA at early and potentially curable stages.

Interestingly, we found an increased proportion of MAC in patients with positive respiratory sample *Aspergillus* culture compared with patients who had negative fungal culture. As previously mentioned, this might be related to the pulmonary background of the patients or due to specific microbial interactions, such as that described between *P. aeruginosa* and *A. fumigatus* in cystic fibrosis (CF) patients (Reece et al., 2018). In a mouse model co-infected with *A. fumigatus* and *M. abscessus*, Monin et al. (2017) reported more inflammatory lesions in the lungs of co-infected animals, which might be due to regulation of Type 1 and Type 17 immune responses. Conversely, analysis of the US Bronchiectasis Research Registry showed no significant increase of *Aspergillus* isolation from the lungs of non CF-fibrosis bronchiectasis patients co-colonized/infected with NTM (21 vs. 16%; *p* = 0.08) (Aksamit et al., 2017). Altogether, the presence of chronic lung diseases or bronchiectasis might facilitate co-colonization and interactions of the pathogens. More studies would be needed to understand whether *Mycobacterium* and *Aspergillus* do interact with each other, resulting possibly in increased colonization and/or worsening of the underlying lung disease.

The major drawback of our study is the lack of a comparison group. Further studies should compare patients co-colonized or infected by both organisms with patients only infected or colonized by one or the other. Another limitation of our study is related to its monocentric design. This study should be extended to other centers to confirm the trends we reported. The rate (28.7%) of patients with a positive mycobacterial culture and a concomitant positive or negative mycological culture was low (28.7%) compare to the rate (79%) of patients with a positive *Aspergillus* spp. culture and a concomitant positive or negative mycobacterial culture. This discrepancy remains unexplained. Due to the retrospective design of the study, physician’ motivations for prescribing both mycobacterial and mycological cultures are unknown. Some clinical and imaging patterns are usually more suggestive of a mycobacterial origin. In that case ruling out TB remains a priority and the question for concomitant *Aspergillus* infection might not be raised. On the contrary, when fungal infection is considered, the question of mycobacterial infection is more systematically addressed. Therefore, and because of the high rate of *Aspergillus* positive cultures concomitant with a positive mycobacterial culture (35,7%), we could recommend being more thorough in considering CPA diagnosis in patients with a positive mycobacterial culture. Systematically adding a mycological culture to respiratory samples positive for *Mycobacteria* could allow the detection of *Aspergillus* colonization subsequently at risk for CPA (Gago et al., 2019). A prospective study should be conducted to address this question.

In conclusion, this is the first study describing challenging cases where *Mycobacterium* and *Aspergillus* are co-isolated but not often responsible for co-infections. Microbial interactions between *Mycobacterium* and *Aspergillus* in the airways may occur but further studies are required to analyze the type of relationship involved.

## Data Availability Statement

All datasets generated for this study are included in the article/Supplementary Material.

## Ethics Statement

Ethical review and approval was not required for the study on human participants in accordance with the local legislation and institutional requirements. Written informed consent for participation was not required for this study in accordance with the national legislation and the institutional requirements.

## Author Contributions

FB, SD, BM, FS, VF, and CA contributed to the conception and design of the research. SD, RL, FF, and FS contributed to the acquisition and analysis of the data. SD performed the statistical analysis. SD, CA, FS, and FB drafted the manuscript. All authors critically revised the manuscript, agreed to be fully accountable for ensuring the integrity and accuracy of the work, and read and approved the final submission version.

## Conflict of Interest

The authors declare that the research was conducted in the absence of any commercial or financial relationships that could be construed as a potential conflict of interest.
