# Supplementary material for: Concomitant Presence of Aspergillus Species and Mycobacterium Species in the Respiratory Tract of Patients: Underestimated Co-occurrence?
Source: Front Microbiol. 2020 Jan 10;10:2980. doi: 10.3389/fmicb.2019.02980 (PMC6967598; doi:10.3389/fmicb.2019.02980)
Supplement: Supplementary file 1 [file Data_Sheet_1.docx]

Supplementary Material

**Supplementary Table S1. Initiation of antimicrobial treatments depending on the isolated *Mycobacterium* or *Aspergillus* species, and the respiratory disorders type.** AMB= Liposomal amphotericin B; Az=Azithromycin; Cl=Clarithromycin; E=Ethambutol; I=Isoniazid; Mo=Moxifloxacin; P=Pyrazinamide; R=Rifampicin; Ri=Rifabutin; VCZ=Voriconazole.

| Species | **Initiation of treatment**  **n (% of patient treated by species or clinical presentation)** | **Treatment**  **(n patients treated with antimicrobial association)** |
| --- | --- | --- |
| ***M. tuberculosis* complex (n=14)** | 14 (100) | IREP |
| **NTM (n=36)** | 12 (33) |  |
| *M. avium* complex (n=19) | 9 (47.3) | RECl (4); REAz (3); RiECl (2) |
| *M. xenopi* (n=8) | 2 (25) | REMo (1); RECl (1) |
| *M. kansasii (n=1)* | 1 (100) | IRE |
|  |  |  |
| ***Aspergillus* (n=50)** | 5 (10) |  |
| CCPA (n=3) | 2 (66) | VCZ |
| Putative IPA (n=1) | 1(100) | VCZ |
| Probable IPA (n=2) | 2 (100) | AMB; VCZ |

Footnote:

NTM: Non-tuberculous *Mycobacterium;* CCPA: chronic cavity pulmonary aspergillosis; IPA: invasive pulmonary aspergillosis.

**Supplementary table S2. Comparison of patient’s characteristics by *Mycobacterium tuberculosis* complex versus non-tuberculous *Mycobacterium* (NTM).** * other bacteria were species isolated only once.

|  | ***M.* complex *tuberculosis* (n=14)**  **n (%)** | **NTM**  **(n=36)**  **n (%)** | ***P*-value** |
| --- | --- | --- | --- |
| *Sex ratio M/F* | 3.7 | 1.25 |  |
| *Age (years)* | 54 | 64 | 0.05 |
| *Inhaled corticosteroids* | 0 (0) | 11 (30) | 0.02 |
|  |  |  |  |
| ***CT characteristics*** |  |  |  |
| *Bronchiectasis* | 6 (43) | 26 (72) | 0.05 |
| *-diffuse* | 1 (7,1) | 14 (39) | 0.04 |
| *-localized* | 5 (36) | 12 (33) | NS |
| *Nodules* | 7 (50) | 11 (30) | NS |
| *Micronodules* | 11 (79) | 25 (69) | NS |
| *Consolidation* | 4 (29) | 10 (28) | NS |
| *Ground glass opacities* | 2 (14) | 1 (3) | NS |
|  |  |  |  |
| ***Other bacterial culture***  *Haemophilus influenzae*  *Pseudomonas aeruginosa*  *Staphylococcus aureus*  *Enterobacter cloacae*  Other bacteria* | 0 (0)  0 (0)  0 (0)  0 (0)  0 (0)  0 (0) | 18 (50)  8 (22)  2 (6)  2 (6)  2 (6)  6 (17) | <0.001 |
|  |  |  |  |
| *Concomitant infection with* Aspergillus | 1 (7) | 6 (17) | NS |
|  |  |  |  |
| *Initiation of anti-mycobacterial therapy* | 14 (100) | 12 (32) | <0.001 |
|  |  |  |  |
| *Number of* Aspergillus *section* Fumigati | 11 (79) | 24 (67) | NS |
|  |  |  |  |
| *Death after one year of follow-up* | 3 (21) | 8 (22) | NS |

**Supplementary Table S3.** **Characteristics of the 3 patients with concomitant detection of *Mycobacterium* sp. and *Aspergillus* sp. and who were treated with both antimycobacterial and antifungal drugs at the time of detection.**

| Characteristics | **Patient 1** | **Patient 2** | **Patient 3** |
| --- | --- | --- | --- |
| **Sex, age (years)** | ♀ 79 | ♀ 66 | ♀68 |
| **Co-isolated organisms** | *A. fumigatus* *sensu stricto*/ *M. tuberculosis* | *A. fumigatus* *sensu stricto* / *M. xenopi* | *A. fumigatus sensu stricto / M. avium* complex |
| **Aspergillosis** | *Putative invasive pulmonary aspergillosis* | *Chronic cavitary pulmonary aspergillosis* | *Probable invasive pulmonary aspergillosis* |
| **Underlying conditions** | Necrotizing vasculitis (multinevritis) + methotrexate-induced ILD | Non severe asthma | Severe dermatomyositis + ILD + previous severe opportunistic infections (fusariosis, CMV) |
| **Immunosupressive therapy** | Corticosteroids (high dose)  Methotrexate | Inhaled corticosteroids | Corticosteroids (low dose)  Methotrexate |
| **CT characteristics** | Consolidation  Nodules | Localized bronchiectasis  Excavated Nodules | Diffuse bronchiectasis  Consolidation  Nodules and micronodules  Pleural effusion |
| **Other mycological tests** | BAL GM = 0.04  Serum GM = 0.07  Serology = NEGATIVE  Precipitins = NEGATIVE | BAL GM = 0.04  Serum GM = 0.05  Serology = POSITIVE  Precipitins = POSITIVE | BAL GM = 0.06  Serum GM = 0.08  Serology = NEGATIVE  Precipitins = NEGATIVE |
| **Treatment in chronological order of prescription (duration)** | VCZ (4 days*)  IREP (6 months) | REMo (5 months)  VCZ (8 months)  RiEMo (1 month)  RiEAz (1 month)  EAz (6 months) | VCZ (19 months)  RiECl (29 months) |
| **Outcome** | Alive at 1 year then lost to follow-up | Alive at 3 years | Alive at 3 years |

Az=Azithromycin; Cl=Clarithromycin; CMV=cytomegalovirus E=Ethambutol; I=Isoniazid; ILD: Interstitial lung disease. Mo=Moxifloxacin; P=Pyrazinamide; R=Rifampicin; Ri=Rifabutin; VCZ=Voriconazole

BAL: Bronchoalveolar lavage; GM: galactomannane;

*: early suspension due to severe cytolysis attributed to VCZ with an alternative diagnosis more convincing than putative IPA.
